# Identification of microR-106b as a prognostic biomarker of p53-like bladder cancers by ActMiR

**DOI:** 10.1038/s41388-018-0367-0

**Published:** 2018-07-03

**Authors:** Eunjee Lee, Ana Collazo-Lorduy, Mireia Castillo-Martin, Yixuan Gong, Li Wang, William K. Oh, Matthew D. Galsky, Carlos Cordon-Cardo, Jun Zhu

**Affiliations:** 1 Department of Genetics and Genomic Sciences, New York, NY USA; 2 Icahn Institute of Genomics and Multiscale Biology, Icahn School of Medicine at Mount Sinai, New York, NY USA; 3 Sema4, a Mount Sinai venture, Stamford, CT USA; 4 Departments of Pathology, Icahn School of Medicine at Mount Sinai, New York, NY USA; 5 Department of Pathology, Champalimaud Centre for the Unknown, Lisbon, Portugal; 6 The Tisch Cancer Institute, Icahn School of Medicine at Mount Sinai, New York, NY USA

## Abstract

Bladder cancers can be categorized into subtypes according to gene expression patterns. P53-like muscle-invasive bladder cancers are generally resistant to cisplatin-based chemotherapy, but exhibit heterogeneous clinical outcomes with a prognosis intermediate to that of the luminal and basal subtypes. The optimal approach to p53-like tumors remains poorly defined and better means to risk-stratify such tumors and identification of novel therapeutic targets is urgently needed. MicroRNAs (miRNAs) play a key role in cancer, both in tumorigenesis and tumor progression. In the past few years, miRNA expression signatures have been reported as prognostic biomarkers in different tumor types including bladder cancer. However, miRNA’s expression does not always correlate well with its activity. We previously developed *ActMiR*, a computational method for explicitly inferring miRNA activities. We applied *ActMiR* to The Cancer Genome Atlas (TCGA) bladder cancer data set and identified the activities of miR-106b-5p and miR-532-3p as potential prognostic markers of the p53-like subtype, and validated them in three independent bladder cancer data sets. Especially, higher miR-106b-5p activity was consistently associated with better survival in these data sets. Furthermore, we experimentally validated causal relationships between miR-106-5p and its predicted target genes in p53-like cell line HT1197. HT1197 cells treated with the miR-106b-5p-specific inhibitor were more invasive while cells treated with the miR-106b-5p-specific mimic were less invasive than corresponding controls. Altogether, our results suggest that miR-106b-5p activity can categorize p53-like bladder tumors into more and less-favorable prognostic groups, which provides critical information for personalizing treatment option for p53-like bladder cancers.

## Introduction

Muscle-invasive bladder cancers (MIBCs) can be categorized into subtypes according to gene expression patterns [1–3]. There are two major intrinsic subtypes, luminal, and basal subtypes. MIBC patients of luminal subtype have better prognosis, but patients of basal subtype have better response to cisplatin-based chemotherapy. Each major subtype can be further divided and response to chemotherapy in each subtype is heterogeneous [4]. Prognostic and/or drug response biomarkers for each subtype are needed to better manage MIBC patients. More specifically, p53-like MIBCs are generally resistant to cisplatin-based chemotherapy, but exhibit heterogeneous clinical outcomes with a prognosis intermediate to that of the luminal and basal subtypes. The optimal approach to p53-like tumors remains poorly defined and better means to risk-stratify such tumors and identification of novel therapeutic targets is urgently needed.

Multiple pathways (cell cycle, apoptosis, cell signaling, and angiogenesis) are altered in MIBC and they interact together to influence patient’s drug response and prognosis. Examining mutations or expression change of one gene at a time cannot capture the complexity of tumor biology. MicroRNAs (miRNAs) can post-transcriptionally regulate a large number of genes [5–7] and have been shown to play a key role in tumorigenesis and tumor progression in many types of cancers [8]. MicroRNAs (miRNAs) are a large class of small, non-coding RNA molecules involved in gene regulation through binding to the 3′-untranslated region of their target mRNAs, resulting in mRNA degradation or translation inhibition. MicroRNAs can act either as oncomirs by targeting tumor suppressors, or as tumor suppressors by targeting oncogenes [8]. In bladder cancer, miRNAs have been shown to impact cell cycle progression, epithelial–mesenchymal transition, cytokine–cytokine receptor interaction, and downstream cancer pathways including phosphoinositol 3-kinase (PI3K)-Akt signaling and mitogen-activate protein kinase signaling pathways [9, 10]. Inactivation of oncogenic miRNAs or restoration of tumor-suppressor may have great potential for cancer treatment. Therefore, miRNAs that play key regulatory roles in cancer progression may have potentials as prognostic markers or novel therapeutics.

Most studies have measured miRNA expression levels in tumor samples to identify aberrant miRNA expression in bladder cancer. However, miRNA expression level is not consistent to its functional activity. There are proteins or RNAs that can mediate the influence of miRNAs on target genes, such as RNA-induced silencing complex [11]. The activity of miRNA might be affected by relative abundance of free miRNA to its target genes [12, 13]. Highly expressed RNAs that contain miRNA-binding sites (i.e., competing endogenous RNA) can depress the activity of miRNAs by reducing the amount of functionally miRNAs available to their target genes. Thus, it is critical to accurately quantify the condition-specific regulatory activity of miRNAs by considering their impact on target genes. And cell-based *in vitro* approaches are developed to measure miRNA activity experimentally [14].

We recently developed a computational method, named *ActMiR*, for explicitly inferring the activity of miRNAs *in vivo* based on the changes in expression levels of target genes [15]. We applied ActMiR to identify prognostic miRNAs in ovarian cancer, gliabolastma, triple-negative breast cancer, ER-positive breast cancers. The prognostic value of miR-500a activity in ER-positive breast cancers has been prospectively validated [16], and the molecular mechanism of miR-500a is elucidated in ER-positive breast cancer cell lines [17]. In bladder cancer, many retrospective studies detected prognostic miRNAs based on its expression level [9]. However, these studies did not account for molecular and clinical differences among MIBC subtypes nor explicitly consider causal regulatory effect of miRNAs on oncogenic or tumor-suppressor targets. Here, we applied *ActMiR* to the bladder cancer data set (BLCA) in The Cancer Genome Atlas (TCGA) to identify key miRNAs that can regulate large numbers of genes in bladder cancer. We demonstrated that our inferred miRNA activity could be further used for identifying functional target genes and prognostic biomarkers. Notably, p53-like bladder cancers exhibit heterogeneous clinical outcomes despite being uniformly classified based on gene expression. We identified miR-106b-5p and miR-532-3p as key tumor suppressors in p53-like bladder cancer based on their inferred activities, whereas expression levels of these miRNAs were not significantly associated with survival. We further demonstrated in multiple independent cohorts that higher miR-106b-5p activity in p53-like bladder cancers was consistently associated with better survival, suggesting miR-106b-5p as a potential novel prognostic biomarker. We experimentally validated the predicted causal relationships between miR-106b-5p and its target genes, and showed that overexpression of miR-106b-5p decreased cell invasiveness, whereas knockdown of miR-106b-5p increased cell invasiveness. Our results suggest that bladder cancer patients in each molecular subtype are still heterogeneous and miRNA-mediated regulatory network can be used for dissecting heterogeneity, identifying novel subtype-specific prognostic markers and therapeutic targets.

## Results

### MicroRNA-mediated regulatory network is cancer subtype specific

**Fig. 1:**
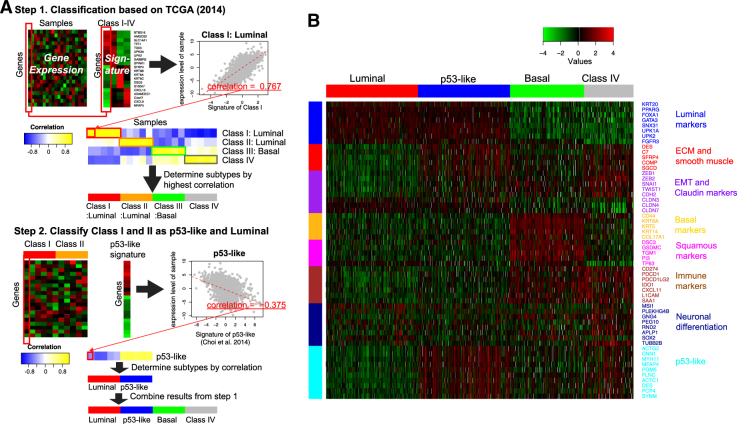
Bladder cancer classification. **a** The overview of classification procedure. We used combined information from two studies [1, 2] to classify bladder tumors into four subtypes: Luminal, p53-like, Basal, and Class IV. First, we classified samples into four groups based on the subtype signatures from TCGA [2] (Step 1). Then, the tumors classified as class I and class II were further divided into Luminal and p53-like subtype based on p53-like signatures [1] (Step 2). **b** The expression levels for selected signature genes in subtypes of bladder cancer samples in TCGA

Owing to its molecular and therapeutic response heterogeneity, many cancers, including bladder cancer, are categorized into subtypes according to gene expression patterns or clinical features [1–3]. Molecular subtypes of bladder cancer have been identified to be associated with distinct clinical features [1–3]. The TCGA [2] and Choi et al. [1] identified four and three molecular subtypes of bladder cancer, respectively. We used combined information from these two studies to classify bladder tumors into four subtypes: luminal, p53-like, Basal, and Class IV (Fig. 1a, Supplementary Table S1, see Materials and Methods for details). Expression levels of luminal markers were high (compared with each marker’s average expression levels) for Luminal and p53-like subtypes, whereas ones of ECM/smooth muscle signature, epithelial–mesenchymal transition (EMT) gene signature, and p53-like markers [1] were high for p53-like subtypes, consistent with the previous observation [3] (the luminal infiltrated subtype was characterized by a wild-type p53 signature) (Fig. 1b). In addition, Basal subtype was characterized by high expression of basal, squamous, and immune markers (Fig. 1b).

The correlation relationships between miRNA and mRNA expression levels were BLCA subtype specific (Supplementary Figure S1), which is consistent with the prior studies [15]. In particular, strong miRNA–mRNA associations based on all samples may be owing to miRNA and mRNA expression level differences among cancer subtypes instead of true causal association between miRNA on mRNA. In such cases, miRNA–mRNA association does not indicate causal and functional connections. These observations suggest that miRNA regulatory mechanisms are cancer subtype specific and each subtype should be studied individually. We studied each subtype individually in all further analyses.

### ActMiR identifies subtype specific key miRNAs

Previously, we developed ActMiR, a method for inferring miRNA activity based on expression levels of miRNAs and their predicted target genes [15]. Supplementary Figure S2 presents an overview of ActMiR method for inferring miRNA activity. In order to obtain robust results [18], we filtered out miRNAs with < 10 target genes, whose activities cannot be accurately inferred using our model.

Applying ActMiR to each subtype of TCGA bladder cancer data set [2], we inferred activity for each miRNA in each sample. To evaluate potential causal relationships between miRNA activities and their correlated mRNAs, we investigated the enrichment of predicted target interactions among genes associated with each miRNA activity. Based on correlation with miRNA activity, more miRNAs whose predicted targets were based on TARGETSCAN [19] were enriched among the genes whose expression levels correlated with miRNA than the results based on correlation with miRNA expression levels (Supplementary Figure S3), for all subtypes, which is similar to the results for other types of cancers [15]. These results suggest that the activity of miRNA implies its functional regulation on target mRNAs levels, and the activity of miRNA can be used to infer key miRNAs, which are defined as miRNAs that causally regulate a large number of mRNAs.

**Fig. 2:**
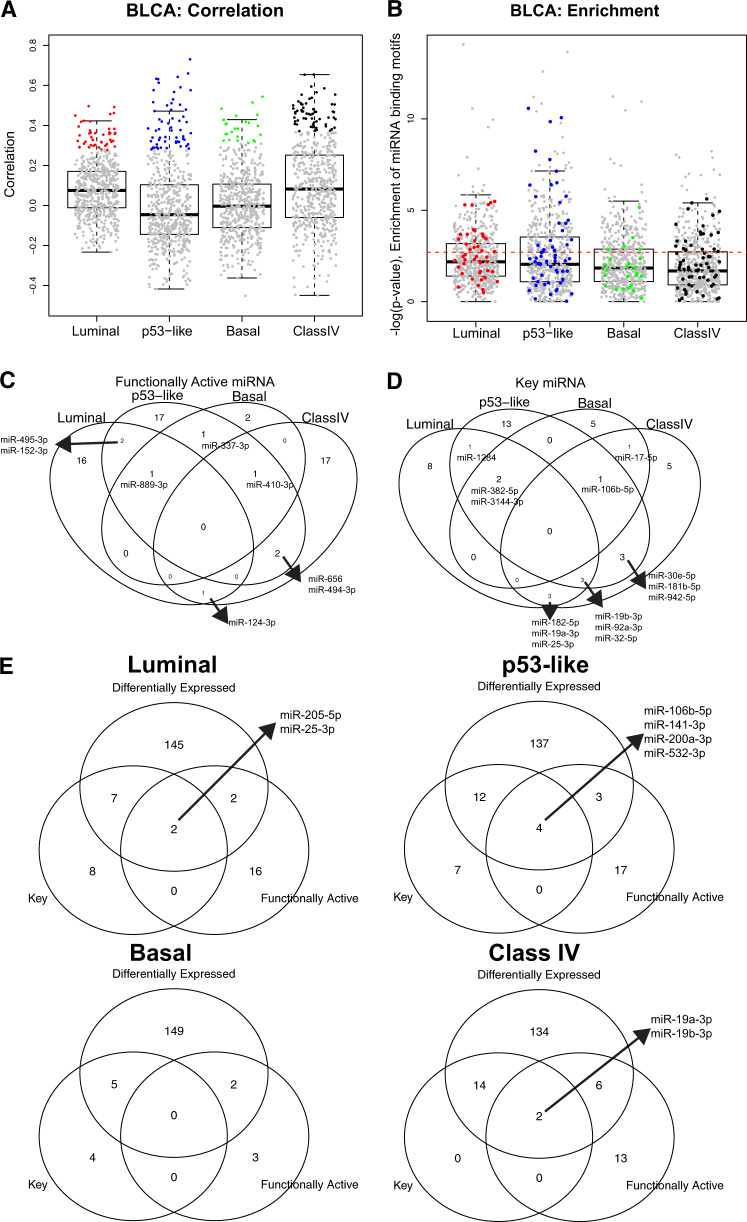
Functionally active and key miRNAs for each subtype. **a** Pearson correlation between expression of miRNA and the inferred activity of miRNAs for each subtype. Boxplot of correlation between inferred activity and expression of miRNAs are shown. The miRNAs with significantly strong correlation at FDR 5% are colored for each subtype. **b** –log(*p* value) of enrichment for miRNA-binding motifs among genes whose expression levels are correlated with each miRNA activity levels. The miRNAs with significantly strong correlation at FDR 5% are colored for each subtype. The colored miRNA whose enrichment is significant at FDR 5% are defined as the functionally active miRNA. **c** The number of functionally active miRNA for each subtype. **d** The number of key miRNAs for each subtype. The key miRNA is defined as the miRNAs whose activity is significantly correlated with a large number of targets’ expression levels. **e** Venn diagram for differentially expressed, key, and functionally active miRNAs for each subtype

We then filtered miRNAs based on three criteria. First, we defined functionally active miRNAs as the miRNAs whose inferred activities and expression levels are positively correlated and whose correlated mRNAs are enriched for miRNAs’ predicted target genes. In general, the correlation between inferred activity and expression level of miRNA was positive corresponding to its role in target degradation, therefore, the distribution of their correlation coefficients is positively skewed (Fig. 2a). Among miRNAs whose inferred activities and expression levels are positively correlated (colored dots in Fig. 2a), we further investigated enrichment for miRNAs’ predicted target genes (Fig. 2b). We detected 20, 24, 5, and 21 functionally active miRNAs for Luminal, p53-like, Basal, and Class IV subtypes, respectively (Fig. 2b, c) at false discovery rate (FDR) 5%, including many subtype-specific miRNAs (Fig. 2c).

Second, we identified key miRNA. To determine key miRNAs, we examined functional target genes whose expression levels are significantly correlated with miRNA activity and are predicted target genes of the miRNA based on TARGETSCAN. Here, we considered only negatively associated genes with miRNA activity. Then, we counted the number of functional target genes for each miRNA activity. We detected 17, 22, 23, and 8 key miRNAs for Luminal, p53-like, Basal, and Class IV subtypes, respectively (Fig. 2d). For example, miR-106b-5p was identified as a key miRNA in three subtypes, Basal, p53-like, and Class IV, and three miRNAs (miR-19b-3p, miR-92a-3p, and miR-32-5p) were identified as key miRNAs in Luminal, p53-like, and Basal subtypes.

Third, we assessed differentially expressed miRNAs compared with normal samples, because we were interested in miRNAs whose functional activity is associated with tumor progression. Among miRNAs considered, 28% of them were differentially expressed compared with normal samples at *P* value < 10^−5^ (corresponds to FDR <1%) by Wilcoxon–Mann–Whitney test (WMW test).

Taken together, we selected miRNAs based on the above criteria, and identified 2, 4 0, and 2 functional key miRNAs that were differentially expressed and functionally active for Luminal, p53-like, Basal, and Class IV subtypes, respectively (Fig. 2e). For example, for p53-like subtype, four miRNAs were identified: i.e., miR-106b-5p, miR-141-3p, miR-200a-3p, and miR-532-3p. Noting that miR-200a-3p and miR-141-3p showed high expression levels for luminal tumors previously [1, 2], the expression levels of these two miRNAs were also high in p53-like subtype (Supplementary Figure S4). Furthermore, the EMT gene ZEB1 and ZEB2 were highly negatively associated with activities of miR-200a-3p and miR-141-3p [20] (*r* = −0.49 and −0.459 and *P* value <1 × 10^−8^ and 1.1 × 10^−7^ for miR-200a-3p, and *r* = −0.484 and −0.480, and *P* value <2 × 10^−8^ and 2.2 × 10^−8^ for miR-141-3p, respectively), indicating potential active role of miR-200 family in the p53-like subtype. These miRNAs were further examined for their prognostic impact.

### ActMiR identifies potential prognostic miRNAs for the p53-like subtype

**Fig. 3:**
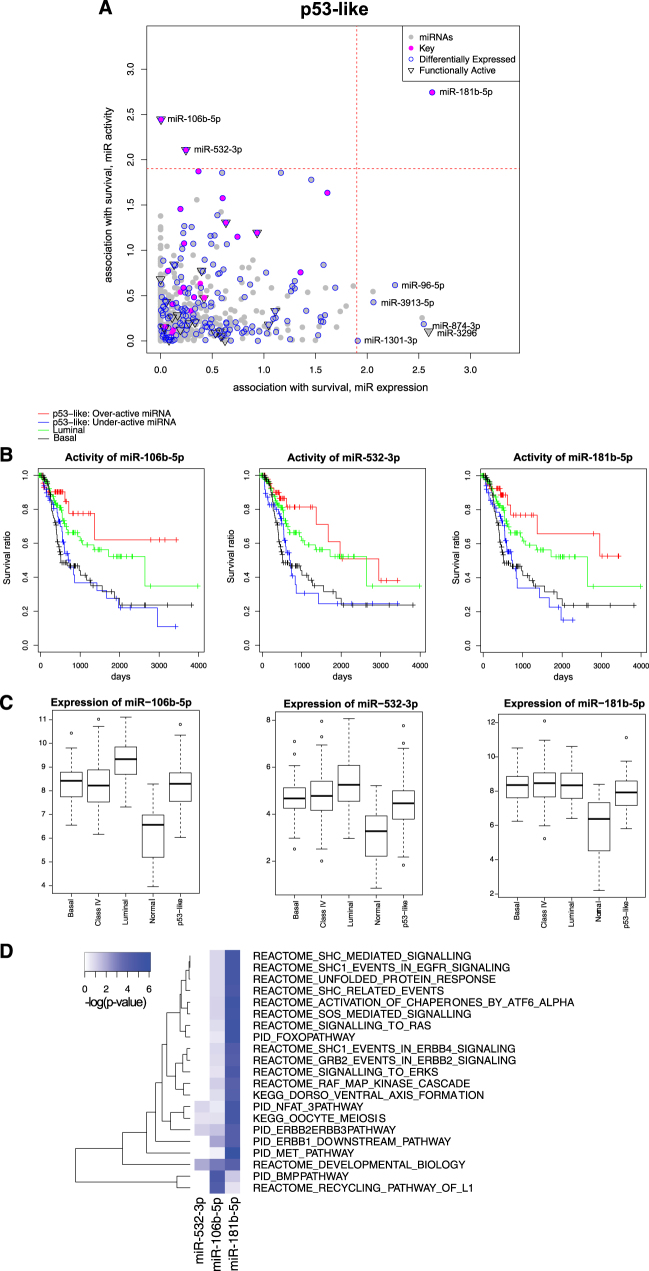
Prognositc key miRNAs of p53-like subtype. **a** Survival analysis based on p53-like subtype of BLCA TCGA samples. Survival prognosis by miRNA activity (*y* axis) and expression level (*x* axis), using a log-rank test was shown. The magenta dot represented key miRNAs, the blue circle represented differentially expressed miRNA, and point down triangle represented functionally active. **b** Kaplan–Meier survival curve based on the activity of miR-106b-5p, miR-532-3p, and miR-181b-5p. The blue and red curve represented under and overactive group among p53-like subtype. The green and black curve represented luminal and basal subtypes, respectively. **c** Expression level of miR-106b-5p, miR-532-3p, and miR-181b-5p for each tumor subtypes and adjacent normal samples. **d** Functional annotation of prognostic and key miRNAs’ functional targets based on canonical pathways. Heatmap of pathway enrichment of functional target genes of each miRNA for p53-like subtype of BLCA TCGA data is shown. The color represented –log(*P* value) of enrichment based on FET. The displayed pathways are significantly enriched for target genes of at least one miRNA

To further explore the functional relevance of the key miRNAs identified above, we tested whether there is association between overall survival and the activity of each miRNA based on each subtype. We identified the activities of 1, 3, 35, and 1 miRNAs associated with survival for Luminal, p53-like, Basal, and Class IV subtypes, respectively (Fig. 3a and Supplementary Figure S5) at 5% FDR (log-rank *P* value <1.2 × 10^−2^). Note that we also tested associations based on the expression of miRNA instead of the activity of miRNA, which resulted in different potential prognostic miRNAs (Fig. 3a and Supplementary Figure S5). For the p53-like subtype 2 (miR-106b-5p and miR-532-3p) of four functional key miRNAs identified were of prognostic significance (Fig. 3a). Tumors with overactive, miR-106b-5p and miR-532-3p were associated with better overall survival rate in p53-like bladder cancers, and their survival was even better than luminal bladder cancers, whereas tumors with under-active miRNAs had similar survival rate as basal bladder cancers (Fig. 3b). In addition, miR-106b-5p and miR-532-3p were differentially expressed compared with normal groups (Fig. 3c) (WMW *P* value <2 × 10^−11^ and 7 × 10^−8^, respectively). Even though the inferred activity and the expression level of miR-106b-5p and miR-532-3p were significantly correlated (*r* = 0.32 and 0.33 and *P* value <3.2 × 10^−4^ and 2.0 × 10^−4^, respectively), their activities were significantly associated with survival, whereas their expression levels were not (Supplementary Figure S6). This result suggests that the activity of miRNA instead of expression level of miRNA is significant in p53-like subtype prognosis. On the other hand, miR-181b-5p’s activity and expression level were not significantly correlated (*r* = 0.10 and *P* value >0.2), indicating it as a non-functionally active miRNA. Even though the activity of miR-181b-5p was most significantly associated with survival, it was not a potential prognostic miRNA for p53-like subtype. We kept miR-181b-5p in our further analyses to demonstrate that activities of functionally active miRNAs are more robust in prognosis predictions.

To understand molecular mechanism underlying prognostic miRNAs, we examined the direct functional target genes of each prognostic miRNA. The functional target genes are defined as genes with predicted miRNA-binding sites whose expression level significantly correlates with inferred miRNA activity at FDR 1%. We predicted 28 functional targets of miR-106b-5p among 3246 differentially expressed genes comparing tumor and normal tissues, and all of the 28 genes negatively correlated with the activity of miR-106b-5p (Fisher’s exact test (FET) *P* value <8.0 × 10^−4^) (Supplementary Table S2). For miR-532-3p and miR-181b-5p, we predicted 15 and 30 functional targets, respectively, and detected that most target genes were negatively correlated with activity miRNA (FET *P* value < 5.2 × 10^−3^ and 1.2 × 10^−3^, respectively). This indicates the role of miRNA in silencing of their cognate target genes by degrading mRNA molecules.

For each prognostic miRNA in the p53-like subtype and miR-181b-5p, we compared its functional target genes with 1320 canonical pathways [21], identifying biological pathways significantly enriched in functional target gene set of each miRNA at FDR <1% (corresponding to FET *P* value <1 × 10^−4^) (Fig. 3d). A total of 21 biological pathways were significantly enriched in target sets of at least one of the three miRNAs (Fig. 3d). Interestingly, the functional target genes of miR-106b-5p were enriched in the bone morphogenetic protein (BMP) pathways. The BMP pathway has been shown to associate with bladder cancer invasiveness and tumor recurrence [22, 23]. McConkey et al. [24] shows bone metastasis occurs disproportionately in p53-like bladder cancer. However, acknowledging the limitations of the clinical annotation in the TCGA BLCA data set, bone metastasis occurred in all subtypes and only 8 out of 27 bone metastasis events reported were in the p53-like subtype. The inferred activity of miR-106b-5p was not associated with bone metastasis status (four and four in miR-106b-5p over- and under-active group, respectively) nor with general metastasis status (8 and 12 in miR-106b-5p over- and under-active group, respectively, FET *P* value <0.23).

### Validation in independent cohort data sets

We aimed to validate our predicted prognostic miRNAs of the p53-like bladder cancer subtype in multiple independent data sets with mRNA expression profiles: Riester et al. [25] including 73 MIBC samples, Lindgren et al. [26] including 131 BLCA samples, and Choi et al. [1] with discovery and validation cohorts including 73 MIBC samples for each cohort. For each data set, we identified 22 out of 73 samples, 45 out of 131 samples, 22 and 21 out of 73 samples, as p53-like subtypes for Riester et al. [25], Lindgren et al. [26], Choi et al. [1] discovery and validation cohorts, respectively (Supplementary Table S1, Supplementary Table S3, and Supplementary Figure S7).

**Fig. 4:**
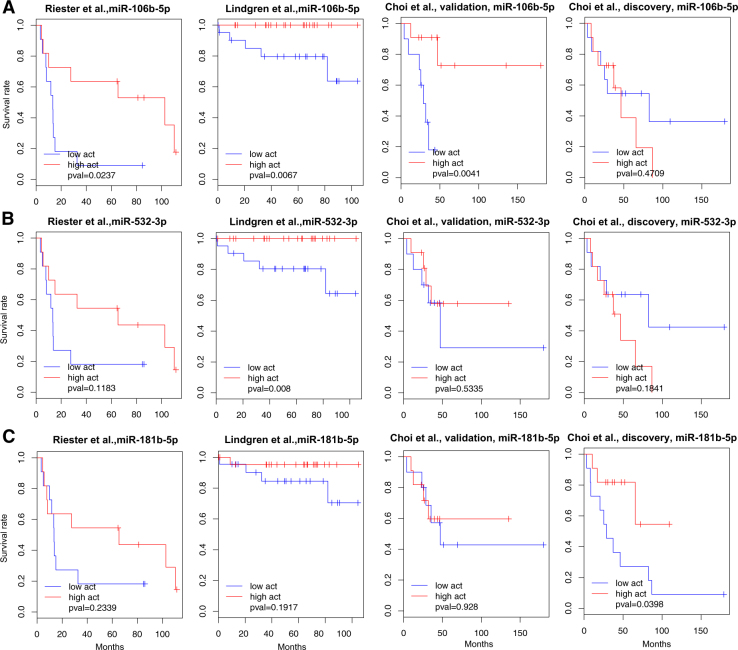
Validation of prognostic key miRNAs of p53-like tumors. **a**–**c** Survival analysis of p53-like tumors in other bladder cancer data sets, Riester et al. [25], Lindgren et al. [26], validation and discovery set of Choi et al. [1]. Kaplan–Meier survival curves based on the activity of miR-106b-5p **a**, miR-532-3p **b**, and miR-181b-5p **c** are shown. The blue and red curve represented under- and overactive group among p53-like subtype

Next, we inferred p53-like specific miRNA activities. To the best of our knowledge, because there is only one bladder cancer study (i.e., the discovery cohort of Choi et al. [1, 27]) with both miRNA and mRNA expression levels for more than 30 samples, we inferred miRNA activity using only mRNA expression levels (See details in Materials and Methods), and then tested association between the inferred activity of miRNA and overall survival. Note that inferred miRNA activities with or without explicitly using miRNA expression level measurement were similar for most of miRNAs in the TCGA bladder cancer data set (see Supplementary Figure S8A). For example, miR-106b-5p showed significantly consistent miRNA activities inferred with and without miRNA expression levels at 1% FDR corresponding to *P* value <1.5 × 10^−4^ (Supplementary Figure S8B, Pearson correlation, *r* = 0.92, *P* value <2.2 × 10^−16^, Bonferroni corrected *α* value <1.2 × 10^−13^) Furthermore, for the discovery cohort of Choi et al. data set [1, 27] with both miRNA and mRNA expression levels, correlations between miRNA activities inferred with and without miRNA expression levels were high for the three miRNAs of interest at 1% FDR corresponding to *P* value <8.5 × 10^−3^ (see Supplementary Figure S8D). We inferred activities of the three miRNAs (miR-106b-5p, miR-181b-5p, and miR-532-3p) in each cohort (Supplementary Table S4). The activity of miR-106b-5p was consistently associated with survival of patients in the three data sets with better survival rate for samples with high miRNA activity (Fig. 4a), whereas the inferred activities of miR-532-3p and miR-181b-5p were associated with survival rate in one of four validation data sets (Fig. 4b, c). In summary, our results suggest the inferred miRNA activity of miR-106b-5p was associated with patient survival in independent validation sets.

### Validating expression of target genes perturbed by the key miRNAs

Inference of miRNA activity using our ActMiR method relies on expression levels of miRNA target genes. To validate causal relationships between functional key miRNAs and their target genes, we perturbed the miRNAs in p53-like bladder cancer cell lines and measured the impact on target genes. First, we selected p53-like bladder cancer cell lines based on two cell lines databases: the Cancer Cell Line Encyclopedia (CCLE) [28] and Cancer Genome Project (CGP) [29]. We classified bladder cancer cell lines and identified three p53-like cell lines in the two databases, and one common p53-like cell line, HT1197, in both database. HT1197 was used for functional experiments to assess miRNA activity through target genes’ expression.

**Fig. 5:**
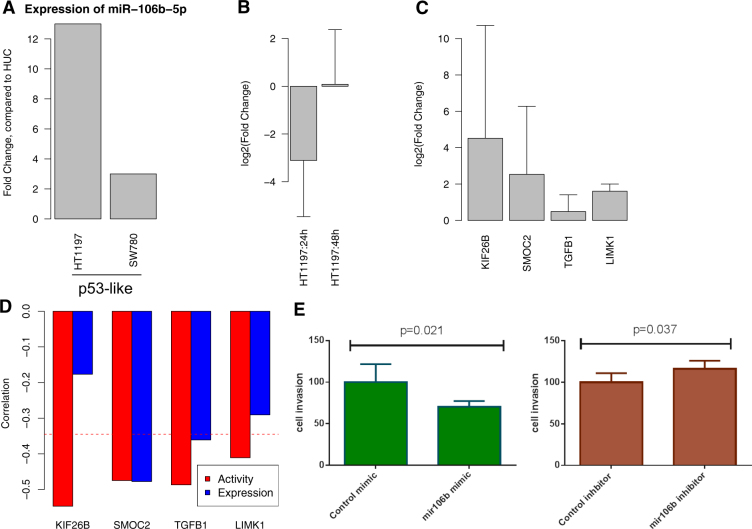
Experimental validations of miR-106b-5p. **a** Expression levels of miR-106b-5p for each cell line. Fold changes compared with HUC are shown for each cell line. **b** log_2_ transformed fold changes after treatment of miR-106b-5p-specific anti-miR inhibitor after 24 and 48 h. **c** The expression changes of target genes after inhibition of miR-106b-5p expression. Comparison of qPCR results of the control and miR-106b-5p inhibitor confirmed that miR-106b-5p regulated the gene expression levels of its predicted target genes. **d** The Pearson correlation between target genes of each miRNAs and miRNA expression (blue) or miRNA activity (red). Even through the expression level of miR-106b-5p did not correlated with the expression levels of their target genes *KIF26B* and *LIMK1*, the inferred miRNA activities of miR-106b-5p significantly correlated with the expression levels of *KIF26B* and *LIMK1*. **e** Cell invasiveness was measured when overexpressing or knockdown miR-106b-5p with the miR-106b-5p-specific mimic or inhibitor relative to corresponding controls in HT1197. The miR-106b-5p-specific inhibitor significantly increased cell invasiveness (*p* = 0.037), whereas the miR-106b-5p-specific mimic significantly decreased cell invasiveness (*p* = 0.021). All error bars indicate 95% confidence Interval

We chose to further investigate miR-106b-5p because it showed consistent prognostic power (Fig. 4a). The expression level of miR-106b-5p was significantly higher in bladder tumor tissues than in normal tissues (*P* value < 2 × 10^−11^, Boneferroni corrected *α* value < 2.08 × 10^−8^, fold-change: 2.63) (Fig. 3c). Similarly, the expression level of miR-106b-5p was higher in p53-like BLCA cell lines than in immortalized urothelial cell line (i.e., Human Urothelium cell (HUC)) (Fig. 5a). The miR-106-5p-specific anti-miR inhibitor decreased expression of miR-106b-5p in HT1197 (i.e., p53-like) after 24 and 48 h treatment (Fig. 5b and Supplementary Figure S9A) relative to anti-miR inhibitor negative controls. We selected 24 h treatment system to measure the expression changes of target genes after inhibition of miR-106b-5p expression. Comparison of qPCR results of the control and miR-106b-5p inhibitor confirmed that miR-106b-5p regulated the gene expression levels of its predicted target genes (Fig. 5c and Supplementary Figure S9B). As expected, *KIF26B*, *SMOC2, TGFB1*, and *LIMK1* mRNA expression increased in anti-miRNA treatment. It is worth to note that *KIF26B*, *TGFB1*, and *LIMK1* were predicted as functional targets of miR-106b-5p only based on inferred miRNAs’ activities but not based on miRNAs’ expression levels (Fig. 5d), further validating the importance of the inferred miRNA activity. On the other hand, the effect of miR-106b-5p on these targets was not valid in basal-like cell line (i.e., 5637 cell, Supplementary Figure S9C and S9D), indicating the subtype-specific effect of miR-106b-5p.

Next, we compared phenotypic changes when overexpressing or knockdown miR-106b-5p with the miR-106b-5p-specific mimic or inhibitor relative to corresponding controls in HT1197 (see Methods, Supplementary Figure S10A and S10B). The miR-106b-5p-specific mimic increased miR-106b-5p expression compared with the control (Supplementary Figure S10B). There was no difference on cell proliferation when treated with the miR-106b-5p-specific mimic or inhibitor relative to corresponding controls in HT1197. When comparing cell invasiveness, the miR-106b-5p-specific inhibitor significantly increased cell invasiveness (*t* test *P* value = 0.037), whereas the miR-106b-5p-specific mimic significantly decreased cell invasiveness (*t* test *P* value = 0.021) (Fig. 5e, Supplementary Table S5), consistent with our observation that high miR-106b-5p activity associated with better survival (Fig. 3b and Fig. 4a).

### Interaction between p53-pathway and miR-106b-5p

We showed that miRNA activities were subtype specific (Fig. 2 and Supplementary Figure S1). MicroRNA miR-106b-5p was active only in the p53-like subtype (Fig. 2e). Here, we investigated the relationship between the activity of miR-106b-5p and p53 signaling pathway in p53-like subtype in multiple ways. First, we checked whether there was regulatory connection between p53 and miR-106b-5p expression or activity. *P53* expression level was not correlated with miR-106b-5p expression (*r* = 0.0543, *P* value = 0.552) nor activity (*r* = −0.0041, *P* value = 0.9634). In addition, *p21* expression level was not correlated with miR-106b-5p expression or activity (*r* = −0.119 and −0.212, *P* value = 0.191 and 0.01881, respectively), either. Next, we collected 39 p53-associated pathway/gene signatures from Molecular Signatures Database (MSigDB) [21] (Supplementary Table S6). We compared miR-106b-5p direct targets (Supplementary Table S2) and p53-related gene sets, and 4 out of 39 p53-associated pathway/gene signatures significantly overlapped miR-106b-5p direct targets, BRUINS_UVC_RESPONSE_VIA_TP53_GROUP_B, MARTINEZ_RB1_AND_TP53_TARGETS_UP, MARTINEZ_TP53_TARGETS_UP, and PID_P53REGULATIONPATHWAY (FET *P* value = 0.00072, 0.0051, 0.0051, and 0.028, respectively). Then, we compared p53-related gene sets and miR-106b-5p-regulated genes. We calculated the correlations between with miR-106b-5p activity and expression levels of all genes measured, and compared correlation distribution of p53-related genes and the rest of genes using non-parametric Kolmogorov–Smirnov (KS) test. Among 39 p53-related gene sets, 5 gene sets significantly overlapped with miR-106b-5p activity correlated genes, MARTINEZ_TP53_TARGETS_UP, MARTINEZ_RB1_AND_TP53_TARGETS_UP, BRUINS_UVC_RESPONSE_VIA_TP53_GROUP_B, MARTINEZ_RB1_AND_TP53_TARGETS_DN, and MARTINEZ_TP53_TARGETS_DN at Bonferroni corrected α-value 0.01 (nominal *P* value = 3.51 × 10^−14^, 1.31 × 10^−13^, 9.30 × 10^−10^, 2.83 × 10^−8^, and 5.09 × 10^−8^, respectively). It is worth to note that p53-related gene sets were enriched for genes negatively correlated with miR-106b-5p activity (Supplementary Figure S11). Taken together, these results suggest that there is an interaction between p53 signaling pathway and miR-106b-5p regulatory network and p53 signaling pathway activation is required for miR-106b-5p to be functional.

### Drug repurposing to identify potential therapeutic treatment for p53-like subtypes

P53-like subtype bladder tumors have been shown to be generally chemotherapy-resistant [1]. Checkpoint blockade immune therapy has been approved for bladder cancers, however, the response rate is only ~20%. We analyzed the recently published metastatic bladder cancer data set including 348 patients [30], grouped patients into molecular subtypes, and inferred miRNA activities in p53-like subtype (Supplementary Table S1, Supplementary Table S3, and Supplementary Figure S7). The response rates to PD-1 blockade were similar for all subtypes (Supplementary Figure S12A), and high miR-106b-5p activity was still associated better survival (Supplementary Figure S12B) in the cohort treated with PD-1 blockade immune therapy. Thus, there is an urgent need to develop therapeutics for p53-like bladder cancer patients, especially for patients with low miR-106b-5p activity. We explored if any existing drug could behave like miR-106b-5p in p53-like subtype bladder cancer (i.e., high miR-106b-5p activity associated with better survival). First, we identified 129 differentially expressed genes according to miR-106b-5p activity at FDR 5%. These include 52 upregulated and 77 downregulated genes for miR-106b-5p overactive group compared with miR-106b-5p under-active group. Then, we identified drugs whose treatment can perturb these differentially expressed genes by using Connectivity Map (CMAP) [31]. We identified emetine that inhibits protein biosynthesis as a potential treatment (Supplementary Table S7). Interestingly, multiple recent studies show that emetine inhibited bladder cancer cell proliferation [32], leukemia cells [33], and ovarian cancer cells [34], suggesting it as a potential novel therapy for p53-like bladder cancers. Furthermore, application of the same procedure to miR-532-3p found emetine as well as wortmannin and LY-294002 that are both PI3K inhibitor as potential treatments, supporting the potential usage of PI3K inhibitors in a subset of bladder cancer [35].

### Factors associated with miR-106b-5p activity within the p53-like subtype

To uncover upstream genetic factors that determine miR-106b-5p activity, we investigated the association between somatic mutation and the activity of miR-106b-5p. We found somatic mutation of intercellular adhesion molecule 1, *ICAM1*, associated with miR-106b-5p activity (WMW *P* value <0.0023, Supplementary Table S8), and the tumors with the mutated gene had lower miR-106b-5p activity than the others. We also identified significant association between *TP53* mutation and the activity of miR-532-3p with the wild-type p53-associated with high activity of miR-532-3p.

We also compared abundance of tumor-infiltrated immune cells and inferred activity of miR-106b-5p, and found no association (Supplementary Figure S13).

## Discussion

Bladder cancers can be divided into different molecular subtypes, each associated with distinct response to chemotherapy and clinical outcome [1–3]. Choi et al. [1, 24] defined the p53-like subtype of bladder cancer and demonstrated that such cancers are generally resistant to cisplatin-based chemotherapy. Furthermore, these investigators demonstrated that p53-like muscle-invasive bladder cancers treated with radical cystectomy had a prognosis intermediate to that of the luminal and basal subtypes. A paradigm is emerging, though further validation is required, in which the preferred strategy for luminal muscle-invasive bladder cancers may be cystectomy alone (given their favorable prognosis), whereas basal muscle-invasive bladder cancers may be best suited for neoadjuvant chemotherapy followed by cystectomy [4]. In this setting, the optimal approach to p53-like tumors remains poorly defined and better means to risk-stratify such tumors and identification of novel therapeutic targets is needed.

To understand heterogeneous outcome within each subtype, we applied a computational approach, *ActMiR*, to the TCGA bladder cancer data set. We identified 2, 4, 0, and 2 functional key miRNAs that were associated with patients’ survival for Luminal, p53-like, basal-like and class IV subtype, respectively. For the p53-like subtype, we identified two key miRNAs, miR-106b-5p and miR-532-3p, associated with patients’ survival, and validated them in independent bladder cancer cohorts. The overall survival of the miR-106b-5p or miR-532-3p overactive group was better than that of the luminal subtype, whereas the survival rate of the under-active group was similar to the basal subtype (Fig. 3b). Then, we validated causal relationships between miR-106-5p and its predicted targets *in vitro*, and showed that miR-106-5p decreased cell invasiveness *in vitro*, which may contribute better prognosis for p53-like bladder cancer patients with higher miR-106-5p activity (Fig. 3b and Fig. 4a). The trend was similar in patients treated with PD-L1 blockade immune therapy (Supplementary Figure S12). Thus, novel therapeutics is urgently needed for patients with risk for poor prognosis. We predicted potential therapeutic candidates that might specifically benefit miR-106b-5p under-active p53-like bladder cancers as well as genomic factors associated with miR-106b-5p under- and overactive p53-like bladder cancers. We note that the effects of these predicted candidates have not been systematically validated in p53-like bladder cancers. Further *in vitro* and *in vivo* experiments are needed to demonstrate their therapeutic values.

In general miRNAs’ inferred activities and expression levels were significant positive correlated while there were many cases where the two were not correlated (Fig. 2a). One of potential explanations of the discrepancy between inferred activities and expression levels of miRNAs is sponge effect of long non-coding RNAs (lncRNAs) that contain miRNA-response elements [36]. These lncRNAs can compete with mRNAs for miRNAs, implying less freely available miRNAs interacting with their mRNA targets. Our ActmiR computed activities only based on protein-coding RNAs, which systematically accounts for sponge effect of lncRNAs and other causes. We identified several potential lncRNA-miRNA interactions; the lncRNA transcript contains computationally predicted target sites of miRNA and its expression levels are significantly negatively associated with miRNA activities. Owing to the RNA-seq poly(A) enrichment strategies, the expression levels based on mRNA-seq cannot fully cover the lncRNAs expression levels [37]. It will be useful to systematically investigate miRNA activities affected by lncRNA-miRNA interaction based on the appropriate method such as Ribo-Zero-Seq.

In summary, we showed that even though these miRNAs are functionally active, expression levels of these miRNAs were not associated with prognosis. We experimentally validated for effect of miR-106b-5p on the predicted target genes, demonstrating that our inferred activities, but not expression, can precisely predict its target genes. Furthermore, we showed that overexpression of miR-106b-5p decreased cell invasiveness and higher miR-106b-5p activity was consistently associated with better survival in p53-like bladder cancers in independent data sets. Taken together, our results underscore the value of examining heterogeneous bladder cancers using a systems biology approach.

## Materials and methods

### TCGA data set and other bladder cancer cohort data sets

We analyzed 405 urothelial bladder cancer samples with both genome-wide mRNA and miRNA expression data from TCGA [2]. We downloaded level 3 mapped and gene-level-summarized for RNA-seq data and level 3 reads per million (RPM) mapped miRNAs for each mature miRNA based on small RNA-seq. RPM + 1 and RPKM + 1 were log transformed and used in our subsequent analysis. We assumed that the sufficient expression level of miRNA is essential to its function. Therefore, we excluded miRNAs whose RPM values equals to 0 for > 85% of samples. This procedure results in 813 out of 1222 miRNA to be considered.

In addition, we downloaded four independent bladder cancer data sets to validate our findings: Riester et al. [25] (*N* = 78, GSE31684), Lindgren et al. [26] (*N* = 131, GSE32549), Choi et al. [1] (*N* = 73 for both discovery and validation data sets, GSE48075 and GSE48276), and Mariathasan et al. [30] (*N* = 348, IMvigor210CoreBiologies R package). Each platform’s probe ID was mapped to the corresponding gene symbol and the expression level were averaged over multiple probes mapped to the same gene symbol. For the discovery data set of Choi et al. [1], we also downloaded miRNA expression data (GSE84525) [27]. The number of samples of all independent data sets we used was summarized in Supplementary Table S1 and the clinicopathologic characteristics of all independent cohort data as well as data normalization methods were shown in Supplementary Table S3.

### Cell line data sets

We downloaded the gene expression data of 1037 cancer cell lines from the CCLE [28], including 27 urinary bladder cancer cell lines. The expression data were processed using Robust Multi-array Average (RMA) and normalized using quantile normalization We also downloaded normalized gene expression data of 789 cancer cell lines including 17 urinary bladder cancer cell lines from the Genomics of Drug Sensitivity in Cancer project (http://www.cancerrxgene.org/) [29]. The corresponding drug sensitivity data was downloaded the same website.

### Classification of bladder cancer tumors and cell lines

We classified tumors and cell lines into four (i.e., Luminal, p53-like, Basal, and Class IV) subtypes by comparing gene expression levels with each subtype-associated gene signatures. First, the previous study classified sample into four groups based on the gene expression levels [2], class I ~ IV. Based on the gene list used for classifying samples [2], we measured each subtype’s signature as the mean expression levels of samples within each subtype. For each sample, we measured the Pearson correlation between expression levels and each subtype’s gene signatures, and classified samples as the subtype with the highest correlation. From previous study [2], the class I and II showed features similar to those of luminal A breast cancers, and class III signature were similar to that of basal-like breast cancer. We call class I and II as Luminal-like class and class III as Basal class. Next, we classified tumors in Luminal-like class (class I and class II) into two groups, Luminal and p53-like. The previous study [1] identified p53-like subtype that has the expressed luminal biomarker, and activated wild-type p53 gene expression signature. Therefore, we clustered Luminal-like samples defined from the previous step as p53-like and Luminal class based on the p53-like signature from the previous study [1]. We calculated Pearson correlation gene expression of samples with the p53-like gene signatures, and assigned p53-like class if they show positive correlation, otherwise assigned Luminal class.

Different subtypes associated with different tumor microenvironment. To investigate relationship between tumor molecular subtype and tumor microenvironment, we downloaded relative stromal fraction (e.g., Stromal score) as well as tumor purity (e.g., ESTIMATE score) among TCGA cohort inferred by ESTIMATE [38], a method that uses gene expression signatures to infer the fraction of stromal and immune cells in tumor bulk tissues. The stromal scores and ESTIMATE scores for the four subtypes in TCGA cohort were different (Supplementary Figure S14). The Luminal subtype had very low stromal score compared with other subtypes (*t* test *P* value < 2 × 10^-16^), whereas the stromal scores of p53-like subtype were not significantly different (*t* test *P* value > 0.69) compared with two other subtypes, Basal and Class IV. The markers for p53-like subtype tumors were expressed much higher in p53-like subtype than in basal subtype, suggesting that the difference cannot be explained by the stromal component alone. Also, the stromal score was not significantly associated with survival in p53-like bladder cancer.

### ActMiR procedure

We previously developed ActMiR [15], a method for inferring miRNA activity based on expression levels of miRNAs and expression levels of their predicted target genes as outlined in Supplementary Figure S2. The main idea is to infer miRNA activity based on the changes in expression levels of target genes by using a regression-based model. We need at least 20 samples in each group to reliably infer miRNA activity.

In brief, the ActMiR requires three-step procedure. First, the “baseline” expression levels of miRNA’s target genes, which correspond to the state where the miRNA had no impact, were estimated for each miRNA. Based on the assumption that the sufficient miRNA concentration is essential for its functional activity, the baseline expression level of the target gene of miRNA are defined as the average expression level of the samples with low miRNA expression level (Supplementary Figure S2A). Second, the “degradation” levels affected by the miRNA are defined as the difference between the observed expression levels of targeted genes for each sample and the baseline expression level (Supplementary Figure S2A). Finally, we fitted a linear model between the degradation levels and baseline expression levels of target genes for each sample, implicating the coefficient from a linear fit as the activity of miRNA (Supplementary Figure S2B). Not all predicted target genes with seed sequences are functionally regulated by miRNAs [39]. To take account for the probability of a predicted target gene being a functional target, we used an iteratively reweighted least squares regression method assuming that the higher anti-correlation between miRNA activity and a gene’s expression level across samples indicates the higher possibility of being a functional target.

### ActMiR code availability

The code of ActMiR is freely available at http://research.mssm.edu/integrative-network-biology/Software.html.

### An ActMiR procedure without miRNA expression levels

We developed a method for estimating miRNA activity when there is only mRNA expression profile available. Based on the expression level of negatively associated functional target genes of each miRNA, we calculated the sum of scaled expression levels for each sample. We defined the baseline samples for each miRNA as the sample with low sum of scaled expression levels of negatively associated functional target genes. We defined the bottom 5% of total samples as baseline samples (thus, we needed at least 20 samples in each subtype to robustly estimate miRNA activities). After defining baseline samples, the procedure to estimate miRNA activity is the same as the standard ActMiR procedure described above.

### Association between clinical data and miRNA activities

We tested whether there is an association between overall survival and the activities of each miRNA using the log-rank test. Samples with miRNA activity higher or lower than median activity were assigned to the over- or under-active group, respectively. The Kaplan–Meier Survival curves were fitted [40] for each group and the equivalence of two curves were tested by log-rank test.

### Determine FDR using random permutation

To determine the statistical significance, we randomly permutated the activity (or expression) of each miRNA for 1000 times, then used the resulting empirical null distribution to compute a false discovery rate (FDR).

### Quantification of miRNAs in bladder cancer cell lines

Bladder cancer cell lines including 5637 for basal subtype, SW780 and HT1197 for p53-like subtype as well as the immortalized normal urothelial cell line (i.e., HUC) were previously purchased from ATCC and maintained in Dr. Cordon-Cardo’s lab. An independent batch of HT1197 cell line was also directly purchased from ATCC. These cell lines were used to *in vitro* quantification of miRNAs. RNA was extracted with the RNeasy Minikit (Qiagen) following manufacturer’s protocol. Quantification of miRNA expression was measured following the Taqman®Small RNA Assays Protocol (Life Technologies) using TaqMan®Universal PCR Master Mix II (2 ×). First, we performed a reverse transcription with 1 µg of total RNA, and this product was then used for quantitative PCR. In brief, RT mastermix was prepared with 100 mM dNTPS, MultiScribe Reverse Transcriptase, 10 × Reverse Transcription buffer, RNAse inhibitor and nuclease-free water. TaqMan Small RNA assays were used against specific miRNAs. The qPCR reaction mix consisted of TaqMan®Small RNA Assay (20 ×), product from the RT reaction, nuclease-free water and TaqMan Universal PCR MasterMIx II (2 ×). The real-time PCR system software with FAM-mode was used for the run and for evaluation of obtained results.

### Validating functional target genes of key miRNAs

HT1197 cells, a p53-like bladder cancer cell line, were cultured in Minimum Essential media supplemented with 10% fetal bovine serum and 1% penicillin/streptomycin. Cells were seeded into six-well plates (2.5 × 105 per well) and cultured overnight. Transfection of mirVAna inhibitors against miR-106 (Life Technologies, cat# AM10067) and negative control were performed. Lipofectamin RNAiMAx reagent (Life Technologies) was used to transfect 10 µM of mirVana inhibitors per well according to the manufacturer’s protocol. After 24 and 48 h incubation, the cells were harvested and total RNA was extracted with the RNeasy Minikit (Qiagen). First, downregulation of miR-106b-5p was confirmed following the Taqman®Small RNA Assays Protocol as described above (Supplementary Figure S10A). The experiments were repeated four times.

To assess expression of predicted target genes, such as *KIF26B*, *SMOC2*, *ZFPM2*, *GPC6*, *TGFB1*, and *LIMK1*, we used quantitative reverse transcriptase PCR to compare expression in control versus the miR-106b-5p downregulated cells. For cDNA synthesis reaction, 1 µg of the same total RNA was used with Taqman Reverse transcription reagents (Life technologies). Quantitative PCR (qPCR) was performed using Power SYBR Green Master Mix (Life Technologies) following manufacturer’s protocol. The primer sequences used for PCR reaction are shown in Supplementary Table S9. The experiments were repeated three times.

### Invasion assay

Cell invasion assay was performed using Corning 96-well transwell insert with 8 µm pores according to manufacturer-provided protocol. Transwell insert was coated with 0.5 × base membrane extract. HT1197 cells were transfected with miR-106b-5p inhibitor (Life Technologies, cat# AM10067), mimic (Life Technologies, cat# MC10067) or corresponding controls (mirVana™ miRNA Inhibitor, Negative Control #1, Life Technologies, cat#4464077; mirVana™ miRNA Mimic, Negative Control #1, Life Technologies, cat#4464058) with lipofectamine RNAiMAX (Life Technologies). The total RNA was extracted to confirm knockdown (Supplementary Figure S10A) and overexpression of miR-106b-5p (Supplementary Figure S10B) with six repeats. Cells were serum starved overnight at 24 h post transfection. A total of 5 × 10^4^ cells were seeded into the transwell insert containing serum free medium with five repeats. The transwell culture insert containing cells was then placed on top of a receiver plate containing complete cell culture medium with 10% fetal bovine serum for 16–17 h to allow cells invade to the receiver plate side. The cells migrated to the bottom of the transwell insert were dissociated from the insert into receiver plate by cell dissociation solution and stained with Calcium AM fluorescent dye. The fluorescent reading of the receiver plate was obtained on a SpectraMax Me microplate reader. The fluorescent reading of control cells was set as 100%. *t* test was applied to detect the differences between treatment and control. One transwell was discarded (Supplementary Table S5) as there was an obvious air bubble between the insert and dissociation solution.

### Interaction between p53-pathway and miR-106b-5p

We investigated the relationship between the activity of miR-106b-5p and p53 signaling pathway in p53-like subtype in three ways. First, we measured the correlation between *p53* or *p21* gene and miR-106b-5p expression or activity. Next, we calculated enrichment between miR-106b-5p direct targets (Supplementary Table S2) and p53-related gene sets by Fisher’s Exact test. We collected 39 p53-associated pathway/gene signatures from Molecular Signatures Database (MSigDB) [21] (Supplementary Table S6). Last, we calculated the correlations between with miR-106b-5p activity and expression levels of all genes measured, and compared correlation distribution of p53-related genes and the rest of genes using non-parametric KS test.

### Detecting small molecules that might be effective to p53-like subtypes with poor prognosis

First, we selected differentially expressed genes. For high-active and low-active groups separated by median miRNA activity, we used WMW test to detect differentially expressed genes. The significance level was computed by random permutation of each sample’s expression levels 500 times, which is *P* value <1 × 10^−4^ corresponding to FDR <5%. Next, based on differentially expressed genes, we determined whether these genes were upregulated or downregulated for tumors with high-active miRNAs. Then, we investigated whether these genes were perturbed by drug treatments by using the tool (http://www.broadinstitute.org/cmap/) from the connectivity map [31]. A positive enrichment score from connectivity map represents the treatment of the drug showed similar expression changes for tumors with high-active miRNA compared with tumors with low-active miRNA.

### Association between activity and somatic mutation status

We downloaded level 2 mutation data from “The Cancer Genome Atlas” data portal. For each gene, we assigned the tumor with mutation as the tumor that has at least one non-silent mutation within gene coding region. We explored the association between mutation and the miRNA activity by following two methods. First, we performed WMW test between mutation status and the continuous activity value of miRNA. We also used FET by transforming continuous activity value of miRNA as binary values, high and low activity. The significant genes with *P* value <0.003 for at least one of two methods were listed in Supplementary Table S8.

### Association between tumor-infiltrating immune cells and miRNA activity

For p53-like TCGA bladder cancer samples, we determined the amount of tumor-infiltrating immune cells by the sum of gene expressions of immune cells for each patient. We used previously defined 547 leukocyte gene markers that distinguish 22 human hematopoietic cell phenotypes [41]. Next, we tested whether the amount of tumor-infiltrating immune cells are associated with miRNA activity by using *t* test based on under-active and overactive group of each miRNA.

## Electronic supplementary material

supplementary materials

## References

[1] Choi W, Porten S, Kim S, Willis D, Plimack ER, Hoffman-Censits J (2014). Identification of distinct basal and luminal subtypes of muscle-invasive bladder cancer with different sensitivities to frontline chemotherapy. Cancer Cell.

[2] Cancer Genome Atlas Research N. (2014). Comprehensive molecular characterization of urothelial bladder carcinoma. Nature.

[3] Robertson AG, Kim J, Al-Ahmadie H, Bellmunt J, Guo G, Cherniack AD (2017). Comprehensive molecular characterization of muscle-invasive bladder cancer. Cell.

[4] Seiler R, Ashab HA, Erho N, van Rhijn BW, Winters B, Douglas J (2017). Impact of molecular subtypes in muscle-invasive bladder cancer on predicting response and survival after neoadjuvant chemotherapy. Eur Urol.

[5] Lee YS, Kim HK, Chung S, Kim KS, Dutta A (2005). Depletion of human micro-RNA miR-125b reveals that it is critical for the proliferation of differentiated cells but not for the down-regulation of putative targets during differentiation. J Biol Chem.

[6] Jansson MD, Lund AH (2012). MicroRNA and cancer. Mol Oncol.

[7] Johnson SM, Grosshans H, Shingara J, Byrom M, Jarvis R, Cheng A (2005). RAS is regulated by the let-7 microRNA family. Cell.

[8] Farazi TA, Hoell JI, Morozov P, Tuschl T (2013). MicroRNAs in human cancer. Adv Exp Med Biol.

[9] Enokida H, Yoshino H, Matsushita R, Nakagawa M (2016). The role of microRNAs in bladder cancer. Investig Clin Urol.

[10] Guancial EA, Bellmunt J, Yeh S, Rosenberg JE, Berman DM (2014). The evolving understanding of microRNA in bladder cancer. Urol Oncol.

[11] Krol J, Loedige I, Filipowicz W (2010). The widespread regulation of microRNA biogenesis, function and decay. Nat Rev Genet.

[12] Ebert MS, Neilson JR, Sharp PA (2007). MicroRNA sponges: competitive inhibitors of small RNAs in mammalian cells. Nat Methods.

[13] Arvey A, Larsson E, Sander C, Leslie CS, Marks DS (2010). Target mRNA abundance dilutes microRNA and siRNA activity. Mol Syst Biol.

[14] Mullokandov G, Baccarini A, Ruzo A, Jayaprakash AD, Tung N, Israelow B (2012). High-throughput assessment of microRNA activity and function using microRNA sensor and decoy libraries. Nat Methods.

[15] Lee E, Ito K, Zhao Y, Schadt EE, Irie HY, Zhu J (2016). Inferred miRNA activity identifies miRNA-mediated regulatory networks underlying multiple cancers. Bioinformatics.

[16] Aushev VN, Lee E, Zhu J, Gopalakrishnan K, Li Q, Teitelbaum SL (2017). Novel predictors of breast cancer survival derived from miRNA activity analysis. Clin Cancer Res.

[17] Degli Esposti D, Aushev VN, Lee E, Cros MP, Zhu J, Herceg Z (2017). miR-500a-5p regulates oxidative stress response genes in breast cancer and predicts cancer survival. Sci Rep.

[18] Harrell F. Regression modeling strategies. as implemented in R package ‘rms’ version. 2013;3(3).

[19] Grimson A, Farh KK, Johnston WK, Garrett-Engele P, Lim LP, Bartel DP (2007). MicroRNA targeting specificity in mammals: determinants beyond seed pairing. Mol Cell.

[20] Gregory PA, Bracken CP, Bert AG, Goodall GJ (2008). MicroRNAs as regulators of epithelial-mesenchymal transition. Cell Cycle.

[21] Subramanian A, Tamayo P, Mootha VK, Mukherjee S, Ebert BL, Gillette MA (2005). Gene set enrichment analysis: a knowledge-based approach for interpreting genome-wide expression profiles. Proc Natl Acad Sci USA.

[22] Kuzaka B, Janiak M, Wlodarski KH, Radziszewski P, Wlodarski PK (2015). Expression of bone morphogenetic protein-2 and -7 in urinary bladder cancer predicts time to tumor recurrence. Arch Med Sci.

[23] Knowles MA, Hurst CD (2015). Molecular biology of bladder cancer: new insights into pathogenesis and clinical diversity. Nat Rev Cancer.

[24] McConkey DJ, Choi W, Shen Y, Lee IL, Porten S, Matin SF (2016). A prognostic gene expression signature in the molecular classification of chemotherapy-naive urothelial cancer is predictive of clinical outcomes from neoadjuvant chemotherapy: a phase 2 trial of dose-dense methotrexate, vinblastine, doxorubicin, and cisplatin with bevacizumab in urothelial cancer. Eur Urol.

[25] Riester M, Taylor JM, Feifer A, Koppie T, Rosenberg JE, Downey RJ (2012). Combination of a novel gene expression signature with a clinical nomogram improves the prediction of survival in high-risk bladder cancer. Clin Cancer Res.

[26] Lindgren D, Sjodahl G, Lauss M, Staaf J, Chebil G, Lovgren K (2012). Integrated genomic and gene expression profiling identifies two major genomic circuits in urothelial carcinoma. PLoS ONE.

[27] Ochoa AE, Choi W, Su X, Siefker-Radtke A, Czerniak B, Dinney C (2016). Specific micro-RNA expression patterns distinguish the basal and luminal subtypes of muscle-invasive bladder cancer. Oncotarget.

[28] Barretina J, Caponigro G, Stransky N, Venkatesan K, Margolin AA, Kim S (2012). The Cancer Cell Line Encyclopedia enables predictive modelling of anticancer drug sensitivity. Nature.

[29] Garnett MJ, Edelman EJ, Heidorn SJ, Greenman CD, Dastur A, Lau KW (2012). Systematic identification of genomic markers of drug sensitivity in cancer cells. Nature.

[30] Mariathasan S, Turley SJ, Nickles D, Castiglioni A, Yuen K, Wang Y (2018). TGF beta attenuates tumour response to PD-L1 blockade by contributing to exclusion of T cells. Nature.

[31] Lamb J, Crawford ED, Peck D, Modell JW, Blat IC, Wrobel MJ (2006). The Connectivity Map: using gene-expression signatures to connect small molecules, genes, and disease. Science.

[32] Foreman KE, Jesse JN, Kuo PC, Gupta GN (2014). Emetine dihydrochloride: a novel therapy for bladder cancer. J Urol.

[33] Moller M, Herzer K, Wenger T, Herr I, Wink M (2007). The alkaloid emetine as a promising agent for the induction and enhancement of drug-induced apoptosis in leukemia cells. Oncol Rep.

[34] Sun Q, Yogosawa S, Iizumi Y, Sakai T, Sowa Y (2015). The alkaloid emetine sensitizes ovarian carcinoma cells to cisplatin through downregulation of bcl-xL. Int J Oncol.

[35] Ching CB, Hansel DE (2010). Expanding therapeutic targets in bladder cancer: the PI3K/Akt/mTOR pathway. Lab Investig.

[36] Ebert MS, Sharp PA (2010). Emerging roles for natural microRNA sponges. Curr Biol.

[37] Zhao W, He X, Hoadley KA, Parker JS, Hayes DN, Perou CM (2014). Comparison of RNA-Seq by poly (A) capture, ribosomal RNA depletion, and DNA microarray for expression profiling. BMC Genomics.

[38] Yoshihara K, Shahmoradgoli M, Martinez E, Vegesna R, Kim H, Torres-Garcia W (2013). Inferring tumour purity and stromal and immune cell admixture from expression data. Nat Commun.

[39] Wu L, Wang Q, Yao J, Jiang H, Xiao C, Wu F (2015). MicroRNA let-7g and let-7i inhibit hepatoma cell growth concurrently via downregulation of the anti-apoptotic protein B-cell lymphoma-extra large. Oncol Lett.

[40] Kaplan EL, Meier P (1958). Nonparametric estimation from incomplete observations. J Am Stat Assoc.

[41] Newman AM, Liu CL, Green MR, Gentles AJ, Feng W, Xu Y (2015). Robust enumeration of cell subsets from tissue expression profiles. Nat Methods.

