# Supplementary material for: Identification of microR-106b as a prognostic biomarker of p53-like bladder cancers by ActMiR
Source: Oncogene. 2018 Jul 3;37(44):5858–72. doi: 10.1038/s41388-018-0367-0 (PMC6212417; doi:10.1038/s41388-018-0367-0)
Supplement: Supplementary file 1 — supplementary materials [file 41388_2018_367_MOESM1_ESM.docx]

***Supplementary Materials for***

**Identification of microR-106b as a prognostic biomarker of p53-like bladder cancer by ActmiR.**

Eunjee Lee^1,2,3^, Ana Collazo Lorduy^4^, Mireia Castillo-Martin^4,5^, Yixuan Gong^6^, Li Wang^1,2,3^, William Oh^6^, Matthew D. Galsky^6^, Carlos Cordon-Cardo^4^, and Jun Zhu^1,2,3,6*^

^1^Department of Genetics and Genomic Sciences, ^2^Icahn Institute of Genomics and Multiscale Biology, Icahn School of Medicine at Mount Sinai, New York, NY, USA; ^3^Sema4, a Mount Sinai venture, Stamford, CT, USA; ^4^Departments of Pathology, Icahn School of Medicine at Mount Sinai, New York, NY, USA; ^5^Department of Pathology, Champalimaud Centre for the Unknown, Lisbon, Portugal; ^6^The Tisch Cancer Institute, Icahn School of Medicine at Mount Sinai, New York, NY, USA;

*To whom correspondence should be addressed.

Contact information for corresponding author: Prof. Jun Zhu, Department of Genetics and Genomic Sciences, Icahn School of Medicine at Mount Sinai, 1425 Madison Avenue room L3-39, New York, NY 10029, USA, Fax: 646-537-8660, Tel:212-659-8942, jun.zhu@mssm.edu

**List of Supplementary Materials:**

Supplementary Figure S1. miRNA-mRNA associations for each subtype of BLCA.

Supplementary Figure S2. Overview ActMiR.

Supplementary Figure S3. The enrichment of miRNA binding motifs based on expression of miRNA and expression of miRNA.

Supplementary Figure S4. Expression levels of miR-200a-3p and miR-141-3p for each subtype (i.e. Luminal, Basal and Class IV) of BLCA TCGA samples.

Supplementary Figure S5. Survival analysis based on each subtype (i.e. Luminal, Basal and Class IV) of BLCA TCGA samples.

Supplementary Figure S6. Kaplan-Meier survival curve based on the expression of miR-106b-5p, miR-532-3p, and miR-181b-5p.

Supplementary Figure S7. Expression levels for selected genes in each subtype.

Supplementary Figure S8. Inferred miRNA activity without miRNA expression levels.

Supplementary Figure S9. Experimental validation in a p53-like cell line HT1197 and in a basal-like cell line 5637.

Supplementary Figure S10. Expression changes treated with the miR-106b-5p specific inhibitor and mimic

Supplementary Figure S11. Cumulative density function plot of the correlation between genes and activity of miR-106b-5p.

Supplementary Figure S12. A metastatic urothelial cancer cohort treated with checkpoint blockade immune therapy.

Supplementary Figure S13. Association between the amount of tumor-infiltrating immune cells and miRNA activity in p53-like bladder cancers.

Supplementary Figure S14. Stromal score (A) and ESTIMATE score (B) by ESTIMATE.

Supplementary Table S1. The number of tumors in each subtype for cohort data sets and cell line data.

Supplementary Table S2. Predicted functional target genes of each miRNA for p53-like subtypes and its expression correlation with miRNA activity.

Supplementary Table S3. Clinicopathologic characteristics of the tumor samples

Supplementary Table S4. Independent cohort data used for validation of survival association with miRNA activity.

Supplementary Table S5. Raw counts for cell invasiveness experiments.

Supplementary Table S6. p53-associated pathway/gene signatures from Molecular Signatures Database (MSigDB)

Supplementary Table S7. The potential drugs that are predicted to be specifically effective to the low miRNA activity group within p53-like subtypes.

Supplementary Table S8. Association between somatic mutation and miRNA activities.

Supplementary Table S9. Primer sequences of target genes for qRT-PCR reaction


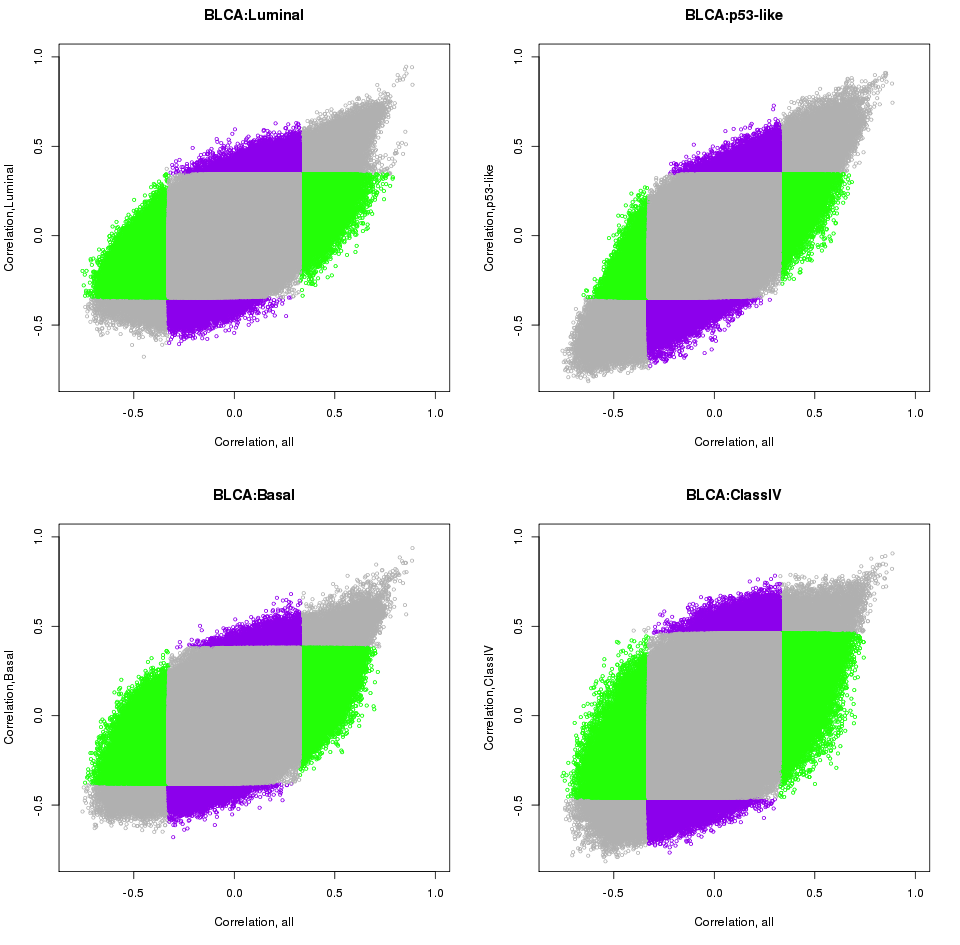


**Supplementary Figure S1. miRNA-mRNA associations for each subtype of BLCA.** All pairwise Pearson correlation between miRNA expression and mRNA expression levels for the subtypes of BLCA. The x-axis represented correlation for each BLCA subtype, and y-axis the correlation of all BCLA samples.


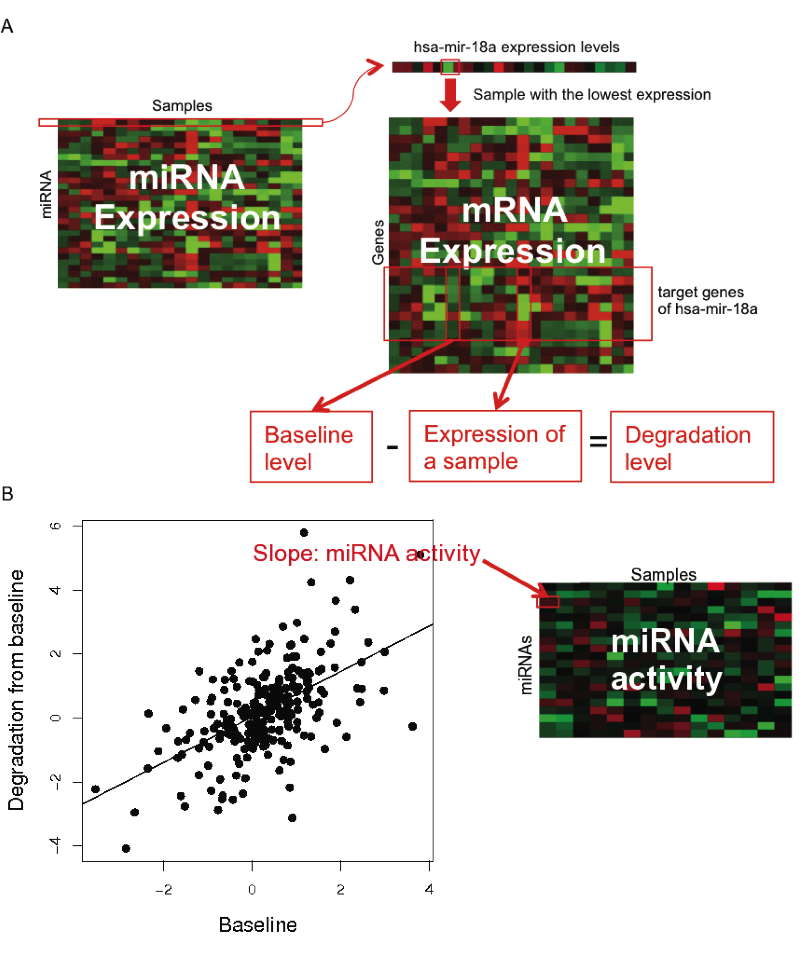


**Supplementary Figure S2. Overview ActMiR (**[**1**](#_ENREF_1)**).** (A) The miRNA and mRNA expression matrix are used to infer the regulatory activity of miRNA for each sample and for each miRNA. For each miRNA, we estimated the baseline expression levels of target genes from their expression levels within samples with the lowest miRNA expression level. For each sample, the degradation levels of target genes were computed as the difference between expression levels in the sample and baseline expression levels. (B) For each sample independently, genome-wide linear regression of baseline expression levels on degradation levels was performed. The coefficient from this linear fitting represents miRNA activity.

**Supplementary Figure S3. The enrichment of miRNA binding motifs based on expression of miRNA and expression of miRNA.** The graph represented –log10 (p-values) of the Fisher’s exact test for enrichment of miRNA binding motifs among genes whose expression levels are correlated with each miRNA expression levels (x-axis) and with each miRNA activity levels (y-axis).

**Supplementary Figure S4. Expression levels of miR-200a-3p and miR-141-3p for each subtype (i.e. Luminal, Basal and Class IV) of BLCA TCGA samples.**

**Supplementary Figure S5. Survival analysis based on each subtype (i.e. Luminal, Basal and Class IV) of BLCA TCGA samples.** Survival prognosis by miRNA activity (y-axis) and expression level (x-axis), using a log-rank test was shown. The magenta dot represented key miRNAs, the blue circle represented differentially expressed miRNA, and point down triangle represented functionally active.

**Supplementary Figure S6. Kaplan-Meier survival curve based on the expression of miR-106b-5p, miR-532-3p, and miR-181b-5p.** The blue and red curve represented under and over active group among p53-like subtype.

**Supplementary Figure S7. Expression levels for selected genes in each subtype.** The expression levels of each cohort for selected genes for labeled gene sets were shown.

**Supplementary Figure S8. Inferring miRNA activity without miRNA expression levels.** (A) Histogram of the Pearson correlation between inferred miRNA activities with and without explicitly using miRNA expression level measurement based on p53-like sample of TCGA bladder cancer data. (B) miR-106b-5p, miR-181b-5p, and miR-532-3p showed consistent miRNA activity with and without miRNA expression levels. (C-D) Same plots as A and B based on the discovery set of Choi et al.([2](#_ENREF_2), [3](#_ENREF_3)) data.

**Supplementary Figure S9. Experimental validation in a p53-like cell line HT1197 (A-B) and in a basal-like cell line 5637 (C-D).** (A) log_2_ transformed fold changes after treatment of miR106-5p specific anti-miR inhibitor after 24h and 48h for HT1197, a p53-like cell line. (B) The expression changes of target genes after inhibition of miR-106b-5p expression in HT1197 cell line. Comparison of qPCR results of the control and miR-106b-5p specific inhibitor are shown for the predicted target genes of miR-106b-5p for HT1197 cell line. (C-D) Same as A and B except using a basal-like cell line 5637 instead of HT1197.

**Supplementary Figure S10. Expression changes treated with the miR-106b-5p specific inhibitor and mimic**. log_2_ transformed fold changes after treatment of miR106-5p specific inhibitor (A) and mimic (B) in HT1197 cell line compared to corresponding controls. The fold change for the miR-106b-5p mimic was larger than the one with the miR-106b-5p specific inhibitor.


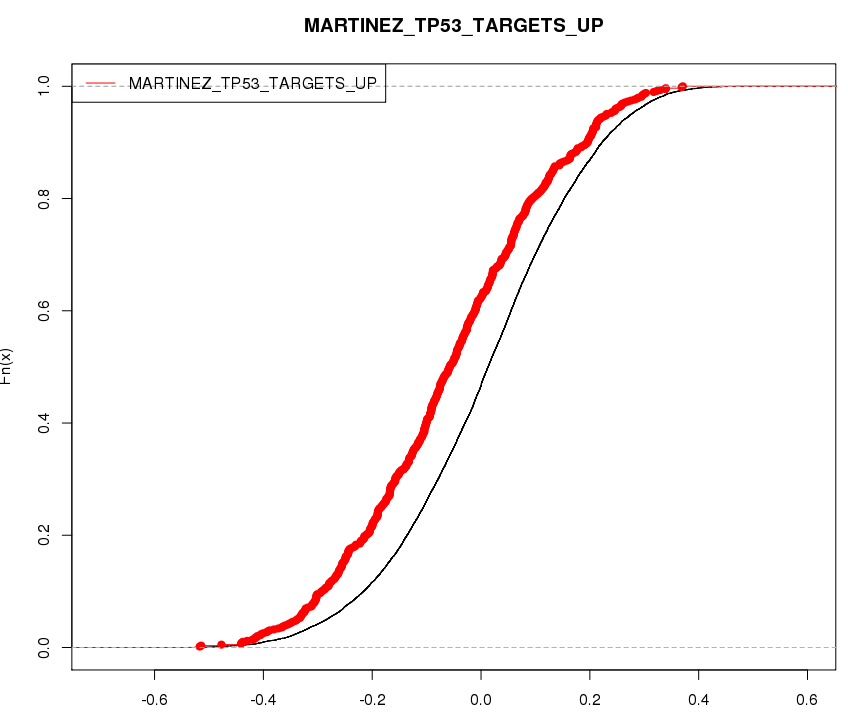


Supplementary Figure S11. Cumulative density function plot of the correlation between genes and activity of miR-106b-5p. The red line represents the genes in the gene set MARTINEZ_TP53_TARGETS_UP and the gray represents the rest of genes.

**Supplementary Figure S12. A metastatic urothelial cancer cohort treated with checkpoint blockade immune therapy**. **A.** Response rates to PD-L1 blockade with atezolizumab in metastatic urothelial cancer ([4](#_ENREF_4)) for each tumor subtype and high/low miR-106b-5p activity groups of p53-like subtype. PD, progressive disease; SD, stable disease; PR, partial response; CR, complete response. **B.** High miR-106b-5p activity was associated better survival.

**Supplementary Figure S13. Association between the amount of tumor-infiltrating immune cells and miRNA activity in p53-like bladder cancer.** The amount of tumor-infiltrating immune cells is defined by the sum of gene expression levels of 547 leukocyte gene markers. The boxplot showed that there is no significant association between the amount of tumor infiltrating immune cells and subtypes based on miRNA activities. *P*-value from t-test is > 0.58, > 0.85, and >0.49 for miR-106b-5p, miR-532-3p, and miR-181b-5p, respectively.

Supplementary Figure S14. Stromal score (A) and ESTIMATE score (B) by ESTIMATE([5](#_ENREF_5)).

**Supplementary Table S1.** The number of tumors in each subtype for cohort data sets and cell line data.

|  | Dataset | Accession # | # Total | # Luminal | # p53-like | # Basal | # Class IV |
| --- | --- | --- | --- | --- | --- | --- | --- |
| Tumor tissue | TCGA |  | 405 | 122 | 122 | 98 | 63 |
|  | Riester et al. ([6](#_ENREF_6)) | GSE31684 | 78 | 17 | 22 | 20 | 19 |
|  | Lindgren et al.([7](#_ENREF_7)) | GSE32549 | 131 | 43 | 45 | 19 | 24 |
|  | Choi et al. ([3](#_ENREF_3)) | GSE48276 | 73 | 21 | 21 | 16 | 15 |
|  |  | GSE48075 | 73 | 20 | 22 | 18 | 13 |
|  | Mariathasan et al.([4](#_ENREF_4)) | IMvigor210CoreBiologies R package | 348 | 112 | 96 | 68 | 72 |
| Cell line | CCLE |  | 27 | 7 | 3 | 9 | 8 |
|  | CGP |  | 17 | 1 | 3 | 6 | 7 |

**Supplementary Table S2.** Predicted functional target genes of each miRNA for p53-like subtypes and its expression correlation with miRNA activity.

| miR-106b-5p | | | miR-532-3p | | |
| --- | --- | --- | --- | --- | --- |
| Genes | Correlation | p-value | Genes | Correlation | p-value |
| KIF26B | -0.546 | 3.5x10^-11^ | DES | -0.636 | 1.7x10^-15^ |
| SMOC2 | -0.474 | 1.6x10^-8^ | PTGIS | -0.576 | 1.7x10^-12^ |
| ZFPM2 | -0.466 | 3.1x10^-8^ | NXPH3 | -0.528 | 1.9x10^-10^ |
| BNC2 | -0.458 | 5.2x10^-8^ | PTGER3 | -0.511 | 8.3x10^-10^ |
| PTGER3 | -0.451 | 9.0x10^-8^ | SYNM | -0.508 | 1.1x10^-9^ |
| GPC6 | -0.446 | 1.2x10^-7^ | MSRB3 | -0.506 | 1.3x10^-9^ |
| TGFB1I1 | -0.429 | 3.8x10^-7^ | DDOST | -0.437 | 2.2x10^-7^ |
| LIMK1 | -0.410 | 1.2x10-^7^ | TPM3 | -0.434 | 2.8x10^-7^ |
| CNN1 | -0.408 | 1.5x10^-6^ | NFIX | -0.392 | 3.9x10^-6^ |
| AHNAK | -0.404 | 1.9x10^-6^ | SLC2A4 | -0.389 | 4.6x10^-6^ |
| MBD5 | -0.402 | 2.1x10^-6^ | TMEM48 | -0.380 | 7.5x10^-6^ |
| MEX3D | -0.399 | 2.6x10^-6^ | NRXN2 | -0.362 | 2.0x10^-5^ |
| CASP2 | -0.394 | 3.3x10^-6^ | ZEB1 | -0.360 | 2.2x10^-5^ |
| PAM | -0.392 | 3.9x10^-6^ | TBL2 | -0.358 | 2.4x10^-5^ |
| ZBTB47 | -0.386 | 5.6x10^-6^ | GRRP1 | 0.347 | 8.9x10^-5^ |
| PSD | -0.385 | 5.9x10^-6^ |  |  |  |
| STX6 | -0.384 | 5.9x10^-6^ |  |  |  |
| CYBRD1 | -0.376 | 9.3x10^-6^ |  |  |  |
| TSHZ3 | -0.372 | 1.2x10^-5^ |  |  |  |
| LASP1 | -0.371 | 1.2x10^-5^ |  |  |  |
| PTPRD | -0.371 | 1.2x10^-5^ |  |  |  |
| SMAD7 | -0.363 | 1.9x10^-5^ |  |  |  |
| NTN1 | -0.360 | 2.2x10^-5^ |  |  |  |
| TNS1 | -0.357 | 2.5x10^-5^ |  |  |  |
| THRA | -0.357 | 2.6x10^-5^ |  |  |  |
| RPS6KA4 | -0.354 | 3.0x10^-5^ |  |  |  |
| FAM129A | -0.351 | 3.5x10^-5^ |  |  |  |
| DPYSL2 | -0.350 | 3.7x10^-5^ |  |  |  |

**Supplementary Table S3.** Clinicopathologic characteristics of the tumor samples

|  | TCGA | | Riester et al. ([6](#_ENREF_6)) | Lindgren et al.([7](#_ENREF_7)) | Choi et al. ([3](#_ENREF_3)) (GSE48276) | Choi et al. ([3](#_ENREF_3)) (GSE48075) | Mariathasan et al.([4](#_ENREF_4)) |
| --- | --- | --- | --- | --- | --- | --- | --- |
| **Gender, *N (%)*** | | | | | | | |
| Female | 106 (26%) | | 21 (27%) | 31 | 14 (19%) | 19 | 76 (22%) |
| Male | 299 (74%) | | 57 (73%) | 100 | 59 (80%) | 54 | 272 (78%) |
| **Age**  **(mean± sd)** | 68.0±10.6 | | 68.9±10.3 | 69.7±10.6 | 65.6±9.8 | 68.7±10.2 | - |
| **Race, *N (%)*** | | | | | | | |
| Asian | 44 (10.8%) | | - | - | 1 (1%) | 0 | 8 (2%) |
| White | 321 (79.2%) | | - | - | 65 (89%) | 54 (74%) | 318 (91%) |
| African American | 23 (5.6%) | | - | - | 3 (4%) | 12 (16%) | 9 (3%) |
| Hispanic | - | | - | - | 4(5%) | 7 (10%) | - |
| **Clinical tumor stage, *N (%)*** | | | | | | | |
| ≤T1 | 2 (0.1%) | | 16 (20%) | 92 (70%) | 11 (15%) | 1 (1%) |  |
| T2 | 122 (30%) | | 51 (65%) | 38 (29%) | 46 (63%) | 41 (56%) |  |
| T3 | 192 (47%) | | 10 (13%) | - | 15 (20%) | 23 (32%) |  |
| T4 | 57 (14%) | | 1 (1%) | - | 1 (1%) | 8 (10%) |  |
| **Clinical lymph node status, *N (%)*** | | | | | | | |
| N0 | 270 (66%) | | 52 (66%) | - | 72 (99%) | 62 (85%) |  |
| N+ | 129 (32%) | | 26 (33%) | - | 1 (1%) | 11 (15%) |  |
| **Positive clinical metastasis, *N (%)*** | | | | | | | |
| M0 | 208 (51%) | | 43 (55%) | - | 71 (97%) | 66 (90%) | - |
| M1 | 11 (2%) | | 35 (45%) | - | 2 (3%) | 7 (10%) | 348 (100%) |
| **Pathologic tumor stage, *N (%)*** | | | | | | | |
| ≤I | 1 (0.2%) | | 0 (0%) | - | 9 (12%) | 10 (18%) | - |
| II | 131 (32%) | | 17 (22%) | - | 13 (18%) | 10 (18%) | - |
| III | 138 (34%) | | 42 (54%) | - | 41 (56%) | 25 (44%) | - |
| IV | 133 (32%) | | 19 (24%) | - | 10 (14%) | 12 (21%) | - |
| **Primary treatment** | | | | | | | |
| Radical  Cystectomy | | - | 78 | - | 47 (64%) | 57 (78%) | - |
| Neoadjuvant Chemotherapy | | 0 | 3 | - | 16 (22%) | 18 (25%) | 272 (78%)  (Platinum) |
| **Median overall survival** (months) | | 17.86 | 31.31 | 53.75 | 35.4 | 30.4 | 10.24 |
| **Expression Platform** | | RNA-seq | Affymetrix | Illumina beadchip | Affymetrix | Illumina beadchip | RNA-seq |
| **Normalization method** | | Reads Per Kilobase of transcript  (RPKM) | GCRMA normalization([8](#_ENREF_8)) | Quantile normalization | Quantile normalization([9](#_ENREF_9)) | Quantile normalization([9](#_ENREF_9)) | Reads Per Kilobase of transcript  (RPKM) |

**Supplementary Table S4.** Independent cohort data used for validation of survival association with miRNA activity.

|  | GEO / ArrayExpress  / R package | Platform | # total | # p53-like | p-value of survival | | |
| --- | --- | --- | --- | --- | --- | --- | --- |
|  |  |  |  |  | miR-106b | miR-181b | miR-532 |
| TCGA |  | RNA-seq | 405 | 122 | 0.0035 | 0.0018 | 0.0078 |
| Riester et al. ([6](#_ENREF_6)) | GSE31684 | Affymetrix | 78 | 22 | 0.0237 | 0.2339 | 0.1183 |
| Lindgren et al.([7](#_ENREF_7)) | GSE32549 | Illumina | 131 | 45 | 0.0067 | 0.1917 | 0.008 |
| Choi et al. ([3](#_ENREF_3)) | GSE48276 | Illumina | 73 | 21 | 0.0041 | 0.928 | 0.5335 |
|  | GSE48075 | Illumina | 73 | 22 | 0.4709 | 0.0398 | 0.1841 |
| Mariathasan et al.([4](#_ENREF_4)) | IMvigor210CoreBiologies | RNA-seq | 348 | 96 | 0.0593 | 0.6833 | 0.0606 |

**Supplementary Table S5**. Raw counts for cell invasiveness experiments.

| Raw counts (FL read) : miR mimic | | | | | | Mean | Variance | *P*-value |
| --- | --- | --- | --- | --- | --- | --- | --- | --- |
| miR mimic control | 68.92606 | 54.25906 | 77.77006 | 48.42306 |  | 62.34 | 13.42 | 0.021 |
| miR-106b-5p mimic | 49.55306 | 46.65406 | 43.22506 | 39.90106 | 39.82206 | 43.83 | 4.26 |  |
| Raw counts (FL read): miR inhibitor | | | | | |  |  |  |
| miR inhibitor control | 332.6888 | 342.4228 | 296.5818 | 256.5238 | 306.3438 | 306.9122 | 33.81 | 0.037 |
| miR-106b-5p inhibitor | 358.4178 | 384.1038 | 341.7678 | 315.0218 | 385.4018 | 356.9426 | 29.74 |  |

**Supplementary Table S6.** p53-associated pathway/gene signatures from Molecular Signatures Database (MSigDB) ([10](#_ENREF_10))

| p53-associated pathway/gene signatures |
| --- |
| KEGG_P53_SIGNALING_PATHWAY  BIOCARTA_P53HYPOXIA_PATHWAY  BIOCARTA_P53_PATHWAY  PID_P53DOWNSTREAMPATHWAY  PID_P53REGULATIONPATHWAY  REACTOME_P53_INDEPENDENT_G1_S_DNA_DAMAGE_CHECKPOINT  REACTOME_P53_DEPENDENT_G1_DNA_DAMAGE_RESPONSE  TANG_SENESCENCE_TP53_TARGETS_UP  TANG_SENESCENCE_TP53_TARGETS_DN  PEREZ_TP53_TARGETS  PEREZ_TP53_AND_TP63_TARGETS  SCHAVOLT_TARGETS_OF_TP53_AND_TP63  AMUNDSON_DNA_DAMAGE_RESPONSE_TP53  CEBALLOS_TARGETS_OF_TP53_AND_MYC_UP  CEBALLOS_TARGETS_OF_TP53_AND_MYC_DN  RAHMAN_TP53_TARGETS_PHOSPHORYLATED  SCIAN_CELL_CYCLE_TARGETS_OF_TP53_AND_TP73_UP  SCIAN_CELL_CYCLE_TARGETS_OF_TP53_AND_TP73_DN  SCIAN_INVERSED_TARGETS_OF_TP53_AND_TP73_UP  SCIAN_INVERSED_TARGETS_OF_TP53_AND_TP73_DN  KANNAN_TP53_TARGETS_UP  KANNAN_TP53_TARGETS_DN  ONGUSAHA_TP53_TARGETS  INGA_TP53_TARGETS  MARTINEZ_TP53_TARGETS_UP  MARTINEZ_TP53_TARGETS_DN  MARTINEZ_RB1_AND_TP53_TARGETS_UP  MARTINEZ_RB1_AND_TP53_TARGETS_DN  AMBROSINI_FLAVOPIRIDOL_TREATMENT_TP53  GALI_TP53_TARGETS_APOPTOTIC_UP  GALI_TP53_TARGETS_APOPTOTIC_DN  MCMURRAY_TP53_HRAS_COOPERATION_RESPONSE_UP  MCMURRAY_TP53_HRAS_COOPERATION_RESPONSE_DN  WU_APOPTOSIS_BY_CDKN1A_VIA_TP53  WU_APOPTOSIS_BY_CDKN1A_NOT_VIA_TP53  STAMBOLSKY_BOUND_BY_MUTATED_TP53  STAMBOLSKY_TARGETS_OF_MUTATED_TP53_UP  STAMBOLSKY_TARGETS_OF_MUTATED_TP53_DN  BRUINS_UVC_RESPONSE_VIA_TP53_GROUP_A  BRUINS_UVC_RESPONSE_VIA_TP53_GROUP_B |

**Supplementary Table S7**. The potential drugs that are predicted to be specifically effective to the low miRNA activity group within p53-like subtypes.

| miRNA | Drug name | Mean | Enrichment | P-value | Specificity | FDR^1^ |
| --- | --- | --- | --- | --- | --- | --- |
| miR-106b-5p | emetine | 0.579 | 0.877 | 0.00034 | 0.0634 | 0.0422 |
|  | tretinoin | 0.23 | 0.405 | 0.0007 | 0.0796 | 0.0652 |
| miR-532-3p | wortmannin | 0.535 | 0.706 | 0 | 0.0065 | 0 |
|  | LY-294002 | 0.305 | 0.381 | 0 | 0.1745 | 0 |
|  | emetine | 0.756 | 0.925 | 0.00004 | 0.0211 | 0.0025 |
|  | cephaeline | 0.681 | 0.915 | 0.00004 | 0.0685 | 0.0025 |
|  | harpagoside | 0.606 | 0.911 | 0.00006 | 0 | 0.0033 |
|  | anisomycin | 0.678 | 0.888 | 0.00016 | 0.0516 | 0.0077 |
|  | levomepromazine | 0.632 | 0.855 | 0.00058 | 0.0195 | 0.0225 |
|  | CP-320650-01 | 0.494 | 0.651 | 0.00084 | 0.0231 | 0.0263 |
|  | thioridazine | 0.319 | 0.423 | 0.00085 | 0.4475 | 0.0263 |
|  | alimemazine | 0.536 | 0.844 | 0.00088 | 0 | 0.0263 |

^1^False discovery rate calculated by Benjamini and Hochberg method.

**Supplementary Table S8.** Association between somatic mutation and miRNA activities.

| miRNA | Gene | p-value from WMW^1^ | t-statistics^2^ | Fisher’s exact test^3^ |
| --- | --- | --- | --- | --- |
| miR-106b-5p | ICAM1 | 0.0022 | 4.99 | 0.0572 |
|  | INTS1 | 0.0027 | -3.18 | 0.0572 |
|  | PHF3 | 0.0228 | -2.31 | 0.0012 |
| miR-532-3p | TP53 | 8.2x10^-5^ | 3.68 | 0.0020 |

^1^p-value from Wilcoxon-Mann-Whitney (WMW) test.

^2^t-statistic from t-test. Positive indicates tumors with mutated status showed high activities of miRNA.

^2^p-value from Fisher’s exact test.

**Supplementary Table S9.** Primer sequences of target genes for qRT-PCR reaction

|  | **Gene** | **primer** | **size** |
| --- | --- | --- | --- |
|  | KIF26B F' | CCTTCTGGATGTAGCGTCGG | 126 |
|  | KIF26B R' | CATTCACCCCGTATTTCTTGCC |  |
|  | SMOC2 F' | AATGACGACGGCACCTACAG | 182 |
|  | SMOC2 R' | GCGGCATCATCTGTTTTTCCT |  |
|  | ZFPM2 F' | CAAACCCCGGCAGATCAAAC | 198 |
|  | ZFPM2 R' | TGGATTCCTTCATCATCACCTTTG |  |
|  | GPC6 F' | CCATGCCTTCTTGGATCGGG | 179 |
|  | GPC6 R' | GATTCTTAAGTGTTCCCCTGCG |  |
|  | TGFB1 F' | CGTGGAGGGGAAATTGAGGG | 98 |
|  | TGFB1 R' | CCGGTAGTGAACCCGTTGATG |  |
|  | LIMK1 F' | CTCCCTTAGACCTCCAGAGC | 107 |
|  | LIMK1 R' | CACTGCAGTCACAACACTTAG |  |

**REFERENCES**

1. Lee E, Ito K, Zhao Y, Schadt EE, Irie HY, Zhu J. Inferred miRNA activity identifies miRNA-mediated regulatory networks underlying multiple cancers. Bioinformatics. 2016;32(1):96-105.

2. Ochoa AE, Choi W, Su X, Siefker-Radtke A, Czerniak B, Dinney C, et al. Specific micro-RNA expression patterns distinguish the basal and luminal subtypes of muscle-invasive bladder cancer. Oncotarget. 2016;7(49):80164-74.

3. Choi W, Porten S, Kim S, Willis D, Plimack ER, Hoffman-Censits J, et al. Identification of distinct basal and luminal subtypes of muscle-invasive bladder cancer with different sensitivities to frontline chemotherapy. Cancer cell. 2014;25(2):152-65.

4. Mariathasan S, Turley SJ, Nickles D, Castiglioni A, Yuen K, Wang Y, et al. TGFbeta attenuates tumour response to PD-L1 blockade by contributing to exclusion of T cells. Nature. 2018;554(7693):544-8.

5. Yoshihara K, Shahmoradgoli M, Martinez E, Vegesna R, Kim H, Torres-Garcia W, et al. Inferring tumour purity and stromal and immune cell admixture from expression data. Nature communications. 2013;4:2612.

6. Riester M, Taylor JM, Feifer A, Koppie T, Rosenberg JE, Downey RJ, et al. Combination of a novel gene expression signature with a clinical nomogram improves the prediction of survival in high-risk bladder cancer. Clinical cancer research : an official journal of the American Association for Cancer Research. 2012;18(5):1323-33.

7. Lindgren D, Sjodahl G, Lauss M, Staaf J, Chebil G, Lovgren K, et al. Integrated genomic and gene expression profiling identifies two major genomic circuits in urothelial carcinoma. PloS one. 2012;7(6):e38863.

8. Wu Z, Irizarry RA, Gentleman R, Martinez-Murillo F, Spencer F. A Model-Based Background Adjustment for Oligonucleotide Expression Arrays. Journal of the American Statistical Association. 2004;99(468):909-17.

9. Ritchie ME, Phipson B, Wu D, Hu Y, Law CW, Shi W, et al. limma powers differential expression analyses for RNA-sequencing and microarray studies. Nucleic acids research. 2015;43(7):e47.

10. Subramanian A, Tamayo P, Mootha VK, Mukherjee S, Ebert BL, Gillette MA, et al. Gene set enrichment analysis: a knowledge-based approach for interpreting genome-wide expression profiles. Proc Natl Acad Sci U S A. 2005;102(43):15545-50. Epub 2005/10/04.
